# In Memoriam: Carol Stroebel

**DOI:** 10.1289/EHP284

**Published:** 2016-05-01

**Authors:** Nsedu Obot Witherspoon

It is with very heavy hearts that the board and staff of the Children’s Environmental Health Network (CEHN) announce the loss of our long-time colleague, champion, and friend Carol Stroebel. Carol, of Winchester, Virginia, passed away surrounded by loved ones on 16 January 2016.

Carol was diagnosed with cholangiocarcinoma (bile duct cancer) in the fall of 2014. Despite this incredible challenge, she remained steadfast in her efforts to protect all children from environmental hazards. By February 2015 she had to step down from her role as CEHN’s Director of Training and Policy to focus on her health. Prior to that role, Carol was a critical consultant to CEHN’s mission, serving as Health Policy Specialist for 14 years.

As a person, Carol was one of the best examples of humanity known. Her genuine kindness and love for others was transparent to all who met her. Carol had a deep passion for the value and protection of life along with equality for all. Her wit, humor, dedication, and heart will be missed by many friends and colleagues.

Carol was a skillful, thoughtful, and intelligent strategist and collaborative partner with the important ability to actually make change happen. Carol had a great passion and desire to truly change the paradigm of how we collectively consider public health, and children’s health specifically, placing children at the center of all that we do. The incorporation of environmental considerations into research questions, training, education, and policy development were all very much a part of her diverse skill set and unique ability to serve as a change agent. The humble and persistent application of strategy that Carol understood so profoundly was, in my opinion, what made Carol remarkable in her work.

Carol’s primary objective was to draw much-needed attention and capacity to children’s vulnerabilities along with providing a range of effective science-based strategies that could be taken to increase their protections. Whether it was work to support and protect the Food Quality Protection Act, the creation and growth of the National Eco-Healthy Child Care^®^ program (http://www.cehn.org/our-work/eco-healthy-child-care/), or the important foundation work that led to the *Blueprint for Protecting Children’s Environmental Health: An Urgent Call to Action* (http://www.cehn.org/our-work/blueprint-for-protecting-childrens-health/), Carol was a true trailblazer. She could have done so many things with the last two decades of her life, but she chose to bless all of us with her passion, expertise, drive, and great intelligence to advance the field of children’s health and push us to only the highest of standards.

Before her work with CEHN, Carol earned a BA in journalism from the University of Wisconsin–Madison. Journalism was her first career, but she quickly gravitated toward public policy work. Serving in government, she worked for outstanding people, most notably Wisconsin Governor Anthony S. Earl and Congressman Norman Mineta, and spent 24 years advocating for highly important issues like highway safety. One of her proudest achievements was her contribution to legislation sponsored by Representative Mineta that resulted in the enactment of the Civil Liberties Act of 1988. That legislation provided redress to Americans of Japanese ancestry who had been interned during World War II, an important step forward for human rights in this country. Carol also served as Director of Intergovernmental Affairs for the National Highway Transportation Safety Administration at the U.S. Department of Transportation during the Clinton administration.

On 12 November 2015 Carol delivered a remarkable and memorable speech as she accepted CEHN’s Child Health Policy Award (which has been renamed in Carol’s honor along with a new CEHN summer internship). Speaking as a life-long advocate of children’s health, she said:

Being a small part of building a new field to help make the paradigm shift for us—to put children in the center, to protect children—and putting my hand to work that fed my soul, it is a great gift. The arc of history is long, we are told, but it bends towards justice, and I have found comfort in that saying, and I suspect maybe a few of you have as well. But in thinking about it I also think that adage can be dangerous. … The arc of history will bend towards justice only if we make it so. We must be aware that we shape this arc every day; where we live, how we live, what we teach our children, even what we buy, affects that arc.

Live as though your life has an impact, Carol told us—because it does. “I didn’t know I was bending history,” she said, “but I made a little contribution, and what a great ride it has been.”

